# A liquid biopsy signature of circulating exosome-derived mRNAs, miRNAs and lncRNAs predict therapeutic efficacy to neoadjuvant chemotherapy in patients with advanced gastric cancer

**DOI:** 10.1186/s12943-022-01684-9

**Published:** 2022-12-12

**Authors:** Ting Guo, Xiao-Huan Tang, Xiang-Yu Gao, Yuan Zhou, Bo Jin, Zi-Qian Deng, Ying Hu, Xiao-Fang Xing, Zi-Yu Li, Jia-Fu Ji

**Affiliations:** 1 Key Laboratory of Carcinogenesis and Translational Research (Ministry of Education/Beijing), Division of Gastrointestinal Cancer Translational Research Laboratory, Peking University Cancer Hospital & Institute, Beijing, 100142 People’s Republic of China; 2 Department of Gastrointestinal Surgery, Peking University Cancer Hospital & Institute, Beijing, 100142 People’s Republic of China; 3 Department of Biomedical Informatics, School of Basic Medical Sciences, Peking University, Beijing, 100191 People’s Republic of China; 4 Department of Clinical Laboratory, Peking University First Hospital, Beijing, 100034 People’s Republic of China; 5 Biological Sample Bank, Peking University Cancer Hospital & Institute, Beijing, 100142 People’s Republic of China

**Keywords:** Exosome, Multi-omics characteristics of RNAs, Advanced gastric cancer, Neoadjuvant chemotherapy, Biomarker panel for efficacy prediction

## Abstract

At present, there is no validated marker to identify the subpopulation of patients with advanced gastric cancer (AGC) who might benefit from neoadjuvant chemotherapy (NACT). In view of this clinical challenge, the identification of non-invasive biomarkers for efficacy prediction of NACT in patients with AGC is imperative. Herein, we aimed to develop a non-invasive, liquid-biopsy-based assay by using an exosome-derived RNAs model based on multi-omics characteristics of RNAs. We firstly used a multi-omics strategy to characterize the mRNAs, microRNAs (miRNAs) and long non-coding RNAs (lncRNAs) profiles of circulating exosome enriched fractions in responders to NACT paired with non-responders, using RNA sequencing. Finally, numerous miRNAs, mRNAs and lncRNAs were identified to be associated with the response to NACT in patients with AGC, and it was validated in an independent cohort with promising AUC values. Furthermore, we established a 6-exosome-RNA panel that could robustly identified responders from non-responders treated with fluorouracil-based neoadjuvant chemotherapy.

**Supplementary Information:**

The online version contains supplementary material available at 10.1186/s12943-022-01684-9.

Gastric cancer (GC) is frequently diagnosed at an advanced (cTNM II-III) stage (AGC), accounting for 71% approximately [1, 2]. Fluorouracil-oxaliplation combination neoadjuvant chemotherapy (NACT) is a routinely therapeutic strategy used for the treatment of patients with AGC and improves survival [1, 3]. However, a significant proportion of AGC patients remain insensitive to NACT [4], and failure to achieve effective treatment responses can risk patients to disease progression, upsurges of tumour burden, toxic side effects and economic losses. Furthermore, responders to NACT have significant benefits on patients’ survival compared to non-responders [5]. Therefore, screening the potential responders to NACT is a critical clinic issue to guide AGC patients to receive the optimal therapeutic strategy. However, no validated markers that could identify the subpopulation of AGC patients who might benefit from NACT have yet been well established.

Recent clinical studies consistently highlight that molecular subtypes of GC are associated with chemotherapeutic efficacy and can be used to guide treatment, such as The Cancer Genome Atlas (TCGA) subtypes [6] and the Asian Cancer Research Group (ACRG) subtypes [7]. In addition, tumour mutation burden (TMB) status was also found to be associated with NACT response [8]. These analyses suggested that NACT treatment should vary according to the molecular characteristics of the tumour. However, multiple endoscopic biopsies to obtain sufficient samples for molecular profiling may cause damage to patients and are difficult to achieve in applications. These findings highlight the urgent need for developing a novel and feasible approach to screen potential responders in the clinic. Liquid biopsy via plasma molecules may be an effective approach to predict responses to NACT in patients with AGC and overcome the above disadvantages.

In contrast to other methods, using exosomes for liquid biopsy offer some distinctive advantages. Exosomes are 30–150 nm extracellular vesicles (EVs) derived from multivesicular bodies (MVBs) that promote cell-cell communication by transmitting cellular molecular constituents, such as DNA, messenger RNAs (mRNAs), non-coding RNAs (ncRNAs), proteins and lipids [9]. Therefore, substantial disease-related molecules can be detected in exosomes. In addition, the protective effects of the exosomal membrane can decrease molecules’ degradation from the enzymes in body liquids, which greatly increase the expressed stability of the exosomal cargos. For example, Min et al. found that exosomal miRNAs used for the early diagnosis of colon cancer have a higher AUC value, compared to plasma miRNAs [10]. Furthermore, liquid biopsy-based on exosomes can achieve multiple acquisitions during treatment and has little damage to patients.

Herein, we firstly used a multi-omics strategy to characterize the mRNAs, microRNAs (miRNAs) and long non-coding RNAs (lncRNAs) profiles of circulating exosome enriched fractions in responders to NACT paired with non-responders, using RNA sequencing. Finally, numerous miRNAs, mRNAs and lncRNAs were identified to be associated with the response to NACT in patients with AGC, and it was validated in an independent cohort with promising AUC values. Furthermore, we established a 6-exosome-RNA panel that could robustly identified responders from non-responders treated with fluorouracil-based neoadjuvant chemotherapy.

## Results and discussion

### Multi-omics characterization of exosomal RNAs correlated with response to NACT in the training sets

A total of 73 individuals including 10 healthy donors, 29 patients responded to NACT and 34 patients who did not respond were enrolled. After plasma collection, all patients received both NACT and surgery (Supplement Fig. 1A). The exosome enriched fractions were extracted from collected plasma by the SEC and ultrafiltration (Supplementary Fig. 1B) method, and these exosomes were confirmed by western blot for specific markers, TEM for morphology and NAT for size distribution (Supplementary Fig. 1C-E). Furthermore, the concentration of total RNA from extracted exosomes enriched fractions was quantitated, but no significant difference was observed among these subgroups (Supplementary Fig. 1F).

**Fig. 1:**
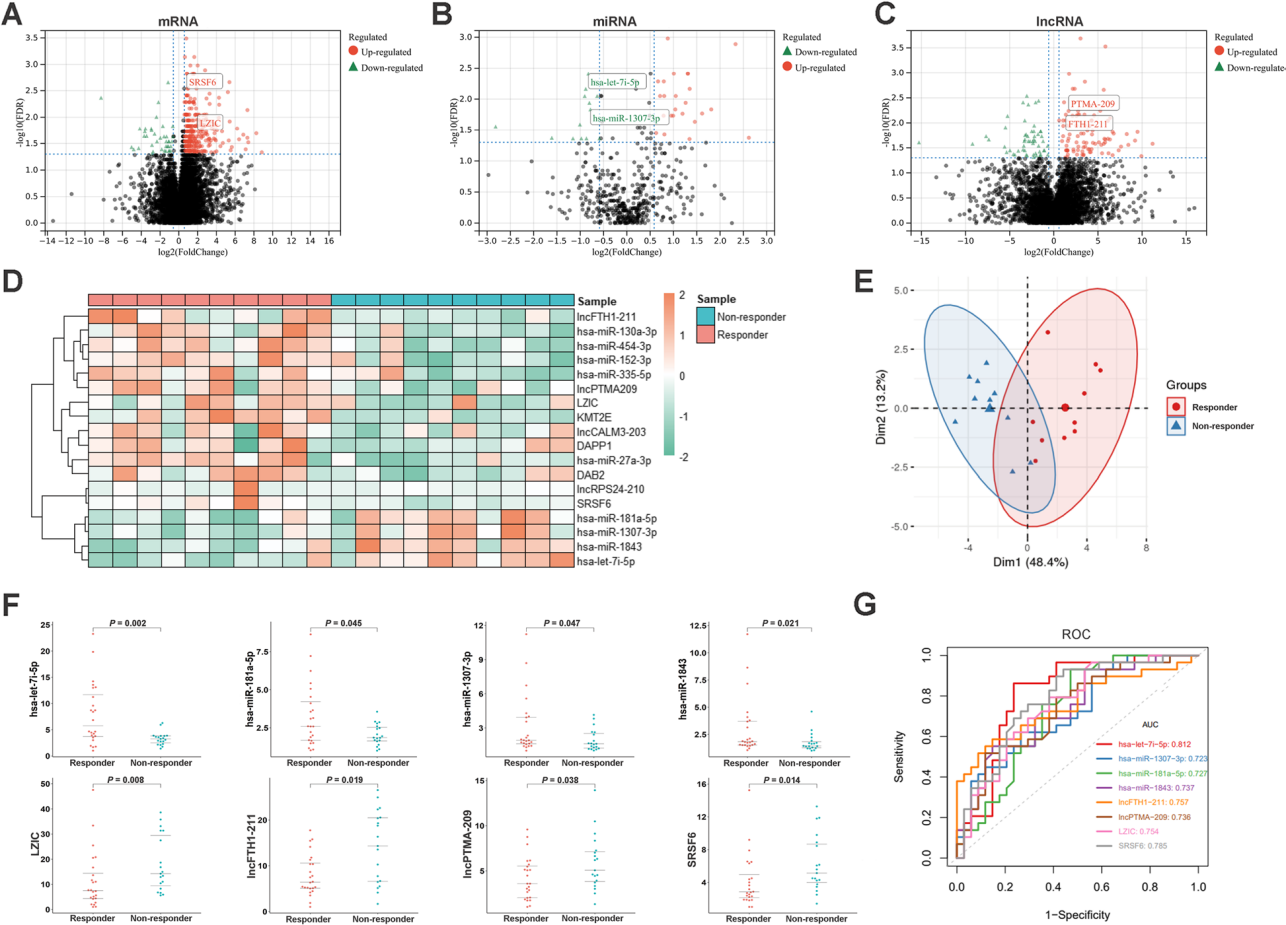
Differential expression analyses and gene expression signature for each group. The differentially expressed mRNAs (**A**), miRNAs (**B**) and lncRNAs (**C**) of plasma-derived exosomes enriched fraction between responders and non-responders to NACT. (**D**) The identified panel of exosomal RNAs including 9 miRNAs, 5mRNAs and 4 lncRNAs, to discriminate potential responders to NACT in AGC patients. (**E**) The PCA method was used to describe gene expression signatures of candidate exosomal RNAs in responders and non-responders. (**G**) The expression levels of 8 verified exosomal RNAs with differential expression and consistent expression trend. (**H**) The ROC analyses of verified 8 exosomal RNAs

Following total RNAs extraction of plasma-derived exosome enriched fractions, multi-omics RNA sequencing was performed to characterize the expression features of mRNAs, miRNAs and lncRNAs in AGC patients with a response or non-response (Fig. 1A-C) to NACT. The intersection of differently expressed RNAs (iDERs) between healthy donors vs patients and responders vs non-responders were used for further analyses to filter data with litter relevance to GC development (Supplementary Fig. 1G). Then, we calculated the sensitivity, specificity and area under the curve (AUC) of each iDERs. Markers with high AUC values (> 0.800), a significant difference (*P* < 0.05, fold-change > 1.5 or < 0.67) and median TPM expression value > 50 were chosen. Finally, a panel of 18 exosomal RNAs, including 5 mRNAs, 9 miRNAs and 4 lncRNAs (Fig. 1D), with separate PCA clusters (Fig. 1E) were identified.

### Validation of the candidate exosome-derived RNAs

To validate the predictive value of plasma exosome-derived candidate RNAs for responders screened in the training sets, the expression levels of these 18 RNAs were detected by qRT-PCR in an independent validation cohort of 43 patients including 19 responders and 24 non-responders. The results showed that expressions of 8 candidate RNAs, including 4 miRNAs (let-7i-5p, miR-181a-5p, miR-1843, miR-1307-3p), 2 mRNAs (LZIC, SRSF6) and 2 lncRNAs (lncFTH1–211, lncPTMA-209), had a significant difference and consistent expression trend with the training set between responders and non-responders (Fig. 1F). Furthermore, we performed ROC analyses and calculated the AUC to verify their potential. Let-7i-5p exhibited the highest AUC of 0.812, while other markers achieved AUC values from 0.723 to 0.785 (Fig. 1G).

### A 6-exosome-RNA biomarker model robustly stratifies efficacy to NACT of patients with AGC

**Fig. 2:**
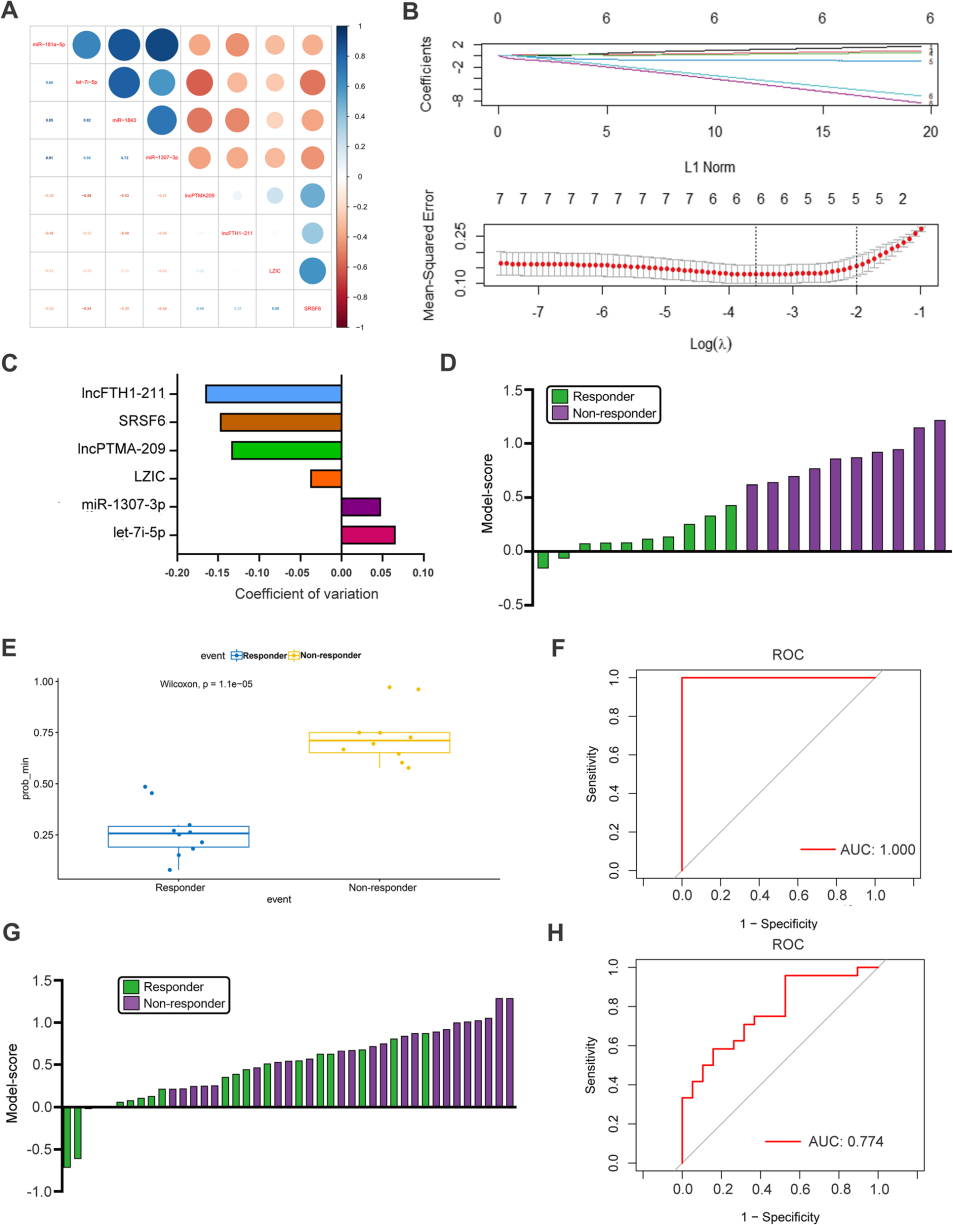
Establishment and validation of a 6-exosome-RNA model. **A** The linear correlation analysis of 8 validated exosomal biomarkers. **B** The most robust predictive genes were identified using the least absolute shrinkage and selection operator (LASSO) Cox regression algorithm. **C** An ensemble of 6 genes remained with nonzero coefficients. **D** The scores of enrolled patients in the training set which were calculated based on the 6-exosome-RNA model. **E-F** The boxplot and the receiver operating characteristic (ROC) analyses illustrated the predictive ability of this 6-exosome-RNA model to identify responders in the training set. **G** The patients’ scores in the validating set calculated according to the 6-exosome-RNA model. **H** The ROC analysis for the predictive power of the focal 6-exosome-RNA model, and the area under the ROC curve was 0.774

To further explore more effective approaches based on these exosomal data, we tried to construct multi-biomarker models to elevate predictive potency. We used the least absolute shrinkage and selection operator (LASSO) Cox regression model to screen the most robust markers for treatment efficacy (Fig. 2A-B). Ten-fold cross-validation was used to overcome over-fitting, and the optimal λ value of 0.1423 was selected. An ensemble of 6 exosomal RNAs (let-7i-5p, miR-1307-3p, LZIC, SRSF6, lncFTH1–211 and lncPTMA-209) remained with individual nonzero coefficients (Fig. 2C), which were integrated to establish a 6-marker predictive signature with an intercept of 0.500. The ability of this exosome-RNA panel to identify responders in the training subset is demonstrated in the bar graph (Fig. 2D). The box graph (Fig. 2E) and ROC analyses (Fig. 2F) showed that this 6-exosome-RNA model identified responders to NACT with a high accuracy (*P* = 0.000011) and AUC value (AUC = 1.00; Specificity: 100%; Sensitivity: 100%).

Subsequently, the accuracy of this 6-exosome-RNA model was further validated in the new cohort with 43 patients. The results found that the scores of responders and non-responder based on our constructed model were significantly different (*P* < 0.05, Fig. 2G). The results of ROC analyses exhibited an AUC of 0.774 (95%CI: 0.64–0.92) in the validating cohort (Fig. 2H).

## Conclusions

Our study indicated that AGC patients who are potential responders to NACT retained specific circulating exosome-derived RNA profiles compared with non-responders. In this study, the predictive ability of 4 plasma exosome-derived miRNAs (let-7i-5p, miR-181a-5p, miR-1843 and miR-1307-3p), 2 mRNAs (LZIC and SRSF6) and 2 lncRNAs (lncFTH1–211 and lncPTMA-209) were identified and validated. Furthermore, we developed a 6-biomarker model that predicted responders with promising sensitivity, specificity and AUC values both in the training and testing cohorts. Our biomarkers and their combination may facilitate individually precision treatment of NACT for patients with AGC to improve treatment efficacy.

## Supplementary Information

**Additional file 1: Supplement Fig. 1.** Plasma collection, exosome separation procedure and identification. (A) Flow diagram to show plasma collection and following treatment. (B) Extraction and sequencing of RNAs from plasma-derived exosome enriched fractions. (C) Detection of exosomal markers by western blot. (D) Exosome morphological detection by transmission electron microscopy. (E) Size distribution of exosomes was assessed by nanoparticle tracking analysis. (F) The RNA concentration of exosomes enriched fraction in different groups. (G) The intersection of differentially expressed RNAs between healthy donors vs AGC patients and responders vs non-responders.

## Data Availability

All data and material can be available from one of the first authors and corresponding authors, who can be contacted on the email address.

## Abbreviations

GC: Gastric cancer
AGC: Advanced gastric cancer
NACT: Neoadjuvant chemotherapy
TCGA: The Cancer Genome Atlas
ACRG: The Asian Cancer Research Group
TMB: Tumour mutation burden
ncRNAs: Non-coding RNAs
mRNA: Messeger RNA
miRNA: MicroRNA
lncRNA: Long non-coding RNA
PKUCH: Peking University Cancer Hospital
EVs: Extracellular vesicles
MVBs: Multivesicular bodies
CT: Computed tomography
cTNM: Clinical tumour-node-metastasis
NAT: Nanoparticle tracking analysis
TEM: Transmission electron microscopy
ROC: Receiver operating characteristic
PCA: Principal component analysis
iDERs: Intersection of differently expressed RNAs
AUC: Area under the curve
