# Supplementary figures and images for: A liquid biopsy signature of circulating exosome-derived mRNAs, miRNAs and lncRNAs predict therapeutic efficacy to neoadjuvant chemotherapy in patients with advanced gastric cancer
Source: Mol Cancer. 2022 Dec 12;21:216. doi: 10.1186/s12943-022-01684-9 (PMC9743536; doi:10.1186/s12943-022-01684-9)

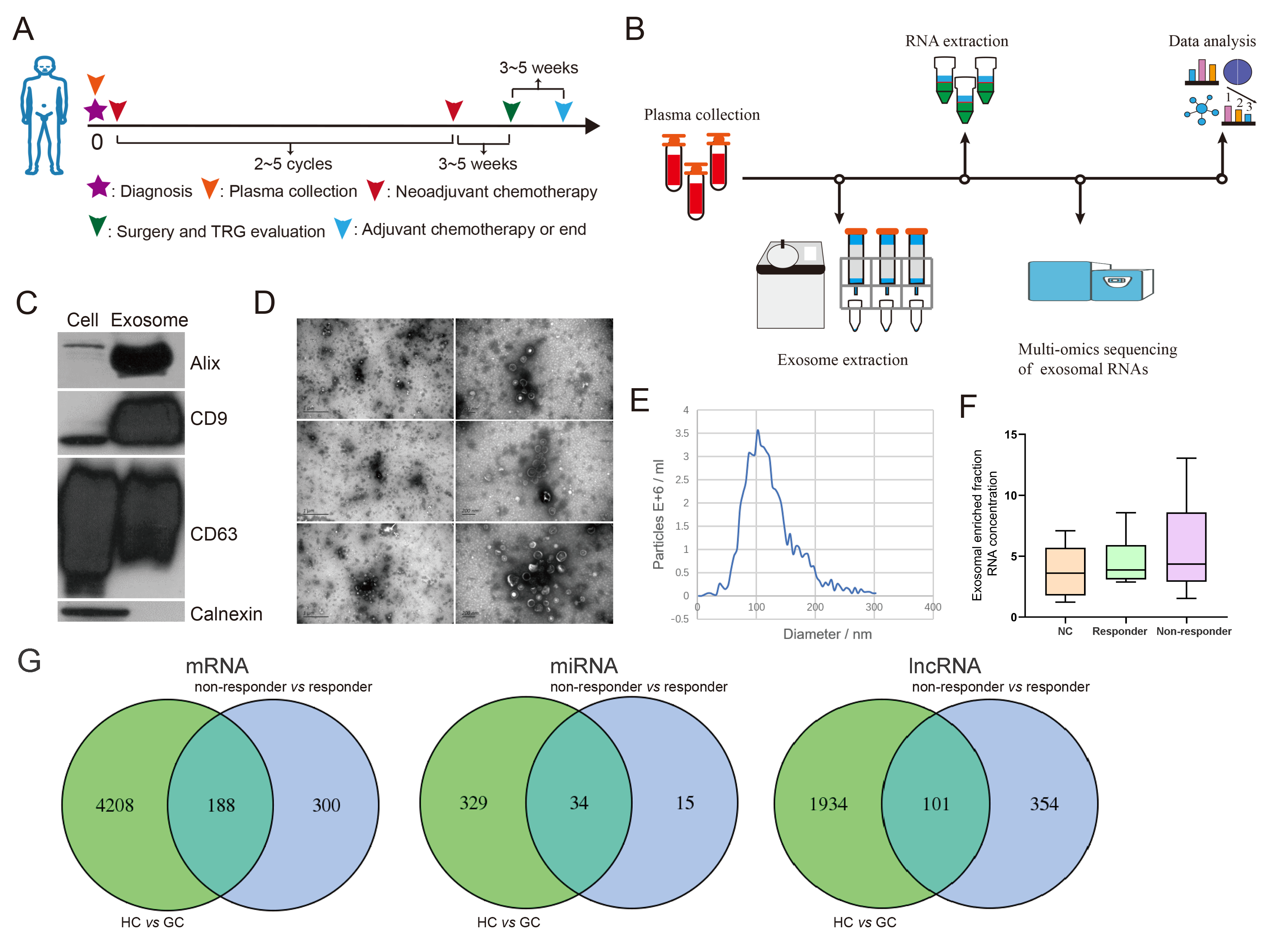

Supplement: Supplementary file 1 — Additional file 1: Supplement Fig. 1. Plasma collection, exosome separation procedure and identification. (A) Flow diagram to show plasma collection and following treatment. (B) Extraction and sequencing of RNAs from plasma-derived exosome enriched fractions. (C) Detection of exosomal markers by western blot. (D) Exosome morphological detection by transmission electron microscopy. (E) Size distribution of exosomes was assessed by nanoparticle tracking analysis. (F) The RNA concentration of exosomes enriched fraction in different groups. (G) The intersection of differentially expressed RNAs between healthy donors vs AGC patients and responders vs non-responders. [file 12943_2022_1684_MOESM1_ESM.tif]
